# Investigation of factors affecting fresh herbage yield in pea (*Pisum arvense* L.) using data mining algorithms

**DOI:** 10.3389/fpls.2024.1482723

**Published:** 2024-11-20

**Authors:** Muhammed İkbal Çatal, Şenol Çelik, Adil Bakoğlu

**Affiliations:** ^1^ Department of Field Crops, Faculty of Agriculture, University of Recep Tayyip Erdogan, Rize, Türkiye; ^2^ Biometry Genetics Unit, Department of Animal Science, Faculty of Agriculture, University of Bingöl, Bingöl, Türkiye; ^3^ Department of Plant and Animal Production, Vocational School of Pazar, University of Recep Tayyip Erdogan, Rize, Türkiye

**Keywords:** CHAID, CART, ANN, MARS algorithm, PEA

## Abstract

This study was carried out to determine the factors affecting the wet grass yield of pea plants grown in Turkey. Wet grass yield was predicted using parameters such as genotype, crude protein, crude ash, acid detergent fiber (ADF), and neutral detergent fiber (NDF) with some data mining algorithms. These techniques provided easily interpretable data trees and precise cutoff values. This led to a comparison of the predictive abilities of data mining methods, including multivariate adaptive regression spline (MARS), Chi-square automatic interaction detection (CHAID), classification and regression tree (CART), and artificial neural network (ANN). To test the compatibility of the data mining algorithms, seven goodness-of-fit criteria were used. The predictive abilities of the fitted models were assessed using model fit statistics such as the coefficient of determination (*R* ^2^), adjusted *R* ^2^, root mean square error (RMSE), mean absolute percentage error (MAPE), standard deviation ratio (SD ratio), Akaike information criterion (AIC), and corrected Akaike information criterion (AICc). With the greatest *R* ^2^ and adjusted *R* ^2^ values (0.998 and 0.986) and the lowest values of RMSE, MAPE, SD ratio, AIC, and AICc (10.499, 0.7365, 0.047, 268, and 688, respectively), the MARS method was determined to be the best model for quantifying plant fresh herbage yield. In estimating the fresh herbage production of the pea plant, the results showed that the MARS method was the most appropriate model and a good substitute for other data mining techniques.

## 1 Introduction

Pea (*Pisum sativum* L.), is an indigenous plant throughout southwest Asia and was among the earliest crops that people farmed, with wild varieties still found in Ethiopia, Afghanistan, and Iran (Maria et al., 2009). According to Berhane et al. (2016), legumes such as peas are essential for crop rotations because they contribute to the breakdown of disease and pest cycles, supply nitrogen, enhance soil microbial activity and multiplicity, improve soil composition, conserve soil water, and provide economic variety. Peas are a cool-season annual crop that fixes nitrogen and has a high ratio of edible protein (23%–33%), along with other biomolecules such as vitamins and carbohydrates (Hafiz et al., 2014).

According to Borreani et al. (2009), field pea, faba bean, and white lupin can all be effectively ensiled with the addition of a lactic acid bacteria inoculum and after a brief wilting period in favorable weather. However, white lupin can only be effectively ensiled with the application of a lactic acid bacteria inoculant due to the low dry matter content at cutting and the quick wilting phase, resulting in a very low dry matter content of wilted and unwilted silages.

When harvested as ensiled feed, legume pulses, including field peas, faba beans, and lupins, are annual crops that are well-suited for brief crop rotations (Borreani et al., 2007). Ensiling pulses as a whole-crop forage provides livestock with less expensive, traceable, and nonanimal-based home-grown protein and starch (Cavallarin et al., 2007). This can also increase the efficiency of the production system in dairy farms by reducing the amount of purchased concentrates fed to the animals (Adesogan et al., 2004).

Forage pea (*Pisum arvense* L.), a highly nutritious and palatable annual legume forage plant, rich in protein within its seeds. After crushing, it can be mixed with roughage. All pea varieties grown in Europe today have flowers in white, green, or yellow colors. Seeds of varieties known in the feed industry in almost all of Europe are evaluated as protein-based feed. If the dried grass of pea is harvested at the appropriate time, it contains about 20% crude protein. Similarly, its seeds contain 20%–30% crude protein, making them a high-quality, nutritious protein source for animals. Peas are used both as dry hay and green seeds for feed, and are valued as a green forage plant in pastures and as green manure to increase nitrogen levels (Özkaynak, 1980; Açıkgöz, 2001).

The aim of this study was to determine the factors affecting green grass yield in pea plants and to predict yield using data on crude ash, crude protein, neutral detergent fiber (NDF), and acid detergent fiber (ADF).

## 2 Materials and methods

### 2.1 Experimental materials

The study was conducted on 14 different pea lines and varieties at the Bingöl University Research and Application Field, located 10 km from the Bingöl city center.

The long-term average temperature in Bingöl province is 12.0°C, whereas in 2015, it was 13.7°C. Similarly, the long-term average maximum temperature is 18.4°C, compared to 19.8°C in 2015. The long-term average minimum temperature is 6.4°C, while in 2015 it was 7.2°C. Annual precipitation also showed a decrease, with a long-term average of 933.9 mm, compared to and 801.8 mm in 2015. These values indicate that 2015 was both warmer and received less rainfall than the long-term averages.

Soil analyses were conducted in the soil analysis laboratory of the Department of Soil Science and Plant Nutrition, Faculty of Agriculture, Bingöl University. The results indicate that the soil has a clayey texture, low organic matter, low salinity, basic pH, deficiency in calcium and potassium, and sufficient phosphorus content.

A field experiment was established in 2015 on a field that had been deep-plowed and tilled with a cultivator and harrow. The experiment followed a randomized block design with three replications. Plot sizes were 5 m in length with 30 cm row spacing, and each plot contained four rows. A seeding rate of 15 kg/ha was used. After planting, sprinkler irrigation was applied to ensure emergence, and weed control was conducted manually throughout the growing season using hand hoeing.

It can be generally accepted that the dependent variable, the wet grass yield of pea plants, is influenced by the genotype predictor variables (crude protein, crude ash, ADF, and NDF).

Crude protein values are shown for each feed. To calculate crude protein, multiply the Kjeldahl nitrogen by either 6.25 or 100/16. On average, proteins contain 16% nitrogen. Crude protein provides little insight into a feed’s true protein and nonprotein composition. Many feed composition charts include digestible protein, but it is more deceptive than crude protein due to the significant contribution of body protein to the apparent protein in feces (Stanton and LeValley, 2006). Crude ash is a proximate chemical composition, similar to crude protein.

Animal digestibility is closely linked to ADF. The availability of net energy from digestible energy and voluntary feed intake are associated with NDF. Both metrics have a stronger correlation with expected animal performance (Stanton and LeValley, 2006).

### 2.2 Statistical methods

#### 2.2.1 Chi-squared automatic interaction detection

For paired-variable assessment, the Chi-squared automatic interaction detection (CHAID) approach may reveal a more trustworthy representation of the unmasked link than either the scatterplot or the smoothed scatterplot. Due to its simplicity in construction, comprehension, and application, CHAID regression tree models are a well-liked approach, particularly among aspiring regression modelers lacking substantial statistical expertise. The foundations of CHAID are also quite appealing: it is an assumption-free technique (i.e., it does not require formal theoretical assumptions to be satisfied) and it is very effective at managing a large number of predictor variables in “big data”. Traditional regression models, on the other hand, are assumption-full, which leaves them vulnerable to unpredictable outcomes and ineffective in handling a large number of predictor variables (Ratner, 2017). Gallagher et al. (2005) state that the CHAID approach is based solely on the classification of categorical dependent variables and uses the Chi-square test to identify categorical independent variables. A representation of the Chi-square test of independence is as follows:


$$\chi^{2}=\sum_{ij}\frac{{\left(O_{ij}-E_{ij}\right)}^{2}}{E_{ij}}$$

where i=1,2,…,r  and   j=1,2,…,

$\text{c}O_{ij}$ : reflects the cell’s observed frequency.

$E_{ij}$ : reflects the cell’s expected frequency.

The CHAID approach consists of three steps: merging, splitting, and stopping (Alkhasawneh et al., 2014).

Continuous variables are converted into ordinal variables before the following algorithm is activated. The mapping of a given *x* into category *C*(*x*) is as follows for a given set of break points $a_{1},a_{2},\ldots ,a_{K-1}$ an (in ascending order):


$$C\left(x\right)=\{\begin{array}{cc} 1 & x\leq a_{1}\\ k+1 & \;a_{k}<x<a_{k+1},\;k=1,\ldots K-2\\ K & a_{K-1}<x \end{array}$$

When estimating the ranks, if *K* is the desired number of bins, $x_{i}$ frequency weights are taken into account for the computation of the break points. The average rank is used if there are ties. The following is an ascending order of the rank and accompanying values: ${\left\{r_{\left(i\right)},x_{\left(i\right)}\right\}}_{i=1}^{n}$

For *k* = 0 to (*K*−1), set $I_{k}=\left\{i:\left[r_{\left(i\right)}\frac{K}{N_{f}1}\right]=k\right\}$ where (*x*) displays the floor integer of *x*. If $I_{k}$ is not empty. $i_{k}=max\left\{i:i\in I_{k}\right\}$ .

In order to exclude the largest, the break points are made equal to the *x* values corresponding to the $i_{k}$ (Breiman et al., 1984; Orhan et al., 2016; Gözüaçık et al., 2018).

#### 2.2.2 Classification and regression tree

Classification and regression tree (CART) is a rule-based, nonparametric machine learning technique that looks for relationships inferred from input characteristics (predictor variables) to target attributes. To improve the accuracy of the target variable prediction, the predictor variable is divided into many areas using this approach (Breiman et al., 1984; Steingberg and Colla, 2016).

Numerous fields, such as agricultural and veterinary sciences, extensive use it (Cak et al., 2013; Eyduran et al., 2013; Çelik and Yilmaz, 2018; Çelik et al., 2018). By locating the primary patterns within the collection of independent variables, the CART technique can be categorize and forecast the values of a specific dependent variable, *Y*. The dependent variable in binary classification problems is binary-valued, while in regression problems, it is continuous or interval-type. The independent variables may be continuous, ordinal, or nominal in nature. Recursive partitioning, the methodical process of building a binary decision tree by dividing each node into two child nodes or not, is the foundation of the CART (Yordanova et al., 2015).

In a regression problem, the mean value of all cases in each terminal node of the decision tree constitutes the projected value. The mean squared error from all variables and threshold values is minimized at each step by determining one independent variable and its suitable threshold value, such as $\theta_{k}$ . For all circumstances where there are two viable answers, “yes” or “no”, the splitting rule has the form $x_{ki}<\theta_{k}$ . In this manner, the independent variable input space is divided into multidimensional, nonoverlapping 2D rectangular or hypercuboid sections. A decision tree is a flow diagram that shows the dependent variable’s categorization and regression prediction models (Yordanova et al., 2015). All beginning cases are dispersed into the regression tree’s terminal nodes.

The following stages can be used to describe the CART method (Gupta et al., 2017):

• The following formal formula is used to calculate the impurity of *D* and the potential result.


$$Gini\left(D\right)=1-\sum_{i}^{m}p_{i}^{2}$$

Where $p_{i}$ is the probability that a tuple in data *D* belongs to class $C_{i}$ , and it is given by $\left|C_{i,D}\right|/\left|D\right|$ .

• The following formula is used to compute each partition attribute’s impurity:


$$Gini_{A}\left(D\right)=\frac{\left|D_{1}\right|}{\left|D\right|}Gini\left(D_{1}\right)+\frac{\left|D_{2}\right|}{\left|D\right|}Gini\left(D_{2}\right)$$

The optimum binary split should then be chosen for use in the following phase by selecting the partition attribute with the lowest Gini index.

• The following formula is utilized to determine the impurity reduction:


$$\Delta Gini\left(A\right)=Gini\left(D\right)-Gini_{A}\left(D\right)$$

The splitting attribute is determined by selecting the feature with the lowest Gini index and the largest drop in impurity (Gupta et al., 2017).

#### 2.2.3 Artificial neural networks

An information processing system called an artificial neural network (ANN) is modeled after biological systems, such the human brain. The brain’s distinctive characteristics include learning new ideas, making judgments, and deriving conclusions from complex and perhaps irrelevant or incomplete data. The widespread use of ANN stems from their limited capacity to mimic brain functions, albeit in a limited capacity (Samarasinghe, 2007). ANNs, therefore, provide an alternative methodology to conventional statistical techniques, which call for the definition of an algorithm and its recording as a computer program. ANNs are instead given example tasks, and their weight coefficients and connections between network parts are automatically adjusted based on the training method (Tadeusiewicz and Lula, 2007).

A typical artificial neuron and the modeling of a multilayered neural network are as follows. The signal flow from inputs $x_{1},x_{2},.,x_{n}$ is considered to be unidirectional, which is a neuron’s output signal flow (*O*). The neuron output signal *O* is given by the following relationship:


$$O=f\left(net\right)=f\left(\sum_{i=1}^{n}w_{i}x_{i}\right)$$

The weight vector in this case is denoted by $w_{i}$ , and the function f(net) is known as an activation (transfer) function. A scalar product of the weight and input vectors defines the variable net,


$$net=w^{T}x=w_{1}x_{1}+w_{2}x_{2}+\ldots +w_{n}x_{n}$$

When *T* is a matrix’s transpose, and the output value *O* is calculated in the most basic scenario as


$$O=f\left(net\right)=\{\begin{array}{c} 1\;\;\;if\;w^{T}x\geq \theta \;\\ 0\;\;\;\;\;otherwise \end{array}$$

where the node type is referred to as a linear threshold unit and θ is known as the threshold level (Abraham, 2005).

##### 2.2.3.1 Perceptron neural networks

By establishing the weights and relevant functions, a multilayer perceptron network can be utilized to solve many exceedingly complex mathematical problems that involve complex nonlinear equations. Different activation functions can be utilized in neurons depending on the kind of issue. In these networks, there are three layers: an input layer that introduces issue inputs, a hidden layer, and an output layer that offers the solution. Backpropagation is a popular training technique for these networks (Manhaj, 2002).

##### 2.2.3.2 Artificial neural network structures

The linked collection of neural networks often uses mathematical techniques to handle data. This multilayer perceptron network (MLP) has three layers: an input layer, a hidden layer, and an output layer for input data, data processing, and output data, respectively. Each layer is composed of many artificial neurons, or nodes. All neurons are linked to each other, except within the same layers. The results are categorized and moved to the output layer using hidden layers. The target variable’s anticipated values are likewise displayed in the output layer. An estimate of the stations’ daily discharge is displayed in the output layer of the current research. The backpropagation technique is used in the multilayer perceptron network’s training process. This algorithm defines the starting weights, which are then assigned to the knots. Next, the model is updated to include the learning samples, after which the output is produced and contrasted with trial samples. When differences exceed the designated cutoff point, the weights are adjusted until the difference between the intended and actual outputs is minimized. This procedure is carried out until the maximum number of iterations or a previously established level of precision is reached (Islam et al., 2001).

The input layer of a feed-forward backpropagation neural network receives external evidence. These inputs are then moved to input variables via the identity transfer function. Through the connections between input layer and hidden layer neurons, scientists were able to access the hidden layers. The basic calculation of ANNs performed in these layers is achieved by connecting weights between the neurons of hidden layers (Nowruzi and Ghassemi, 2016). In order to weight the summations of the outputs from the preceding layer in the neurons of the hidden layer, they are adding biasedly. This total is then transferred using a transfer function. For a neuron in the buried layer, the hyperbolic tangent sigmoid transfer function is implemented by


$$n_{j}=\frac{2}{1+e^{-2Z}}-1$$

where *Z* will be ascertained as follows, and *n_j_* represents the *j*th neuron output.


$$Z=\sum_{i=1}^{r}w_{ij}p_{i}+b_{j}$$

Here, *p_i_* is the *i*th neuron’s output, and *ω_ij_* are the *i*th neuron’s connectivity weights from the previous layer to the *j*th neuron. Furthermore, *b_j_* is the bias, and *r* is the number of neurons in the preceding layer.

In addition to hyperbolic tangent activation, other activation functions such as linear activation function, sigmoid function, exponential linear unit, and Softmax function can be used in artificial neural networks.

The linear activation function can be defined as:


F(Z)=aZ

Any constant value that the user selects can be the value of variable *a* (Sharma et al., 2020).

The sigmoid function can be defined as follows (Sibi et al., 2013):


$$sig\left(Z\right)=\frac{1}{1+e^{-Z}}$$

Exponential linear unit introduces a parameter slope for the negative values of *x*. It uses a log curve for defining the negative values (Sharma et al., 2020).


f(Z)=Z,   Z≥0

$$f\left(Z\right)=a\left(e^{Z}-1\right),\;\;Z<0$$

The Softmax function is a combination of multiple sigmoid functions. Since a sigmoid function is known to yield values between 0 and 1, these values may be interpreted as the probability of the data points belonging to a certain class. The Softmax function can be used for multiclass classification issues, in contrast to sigmoid functions, which are utilized for binary classification. The function yields the probability for each data point across all classes. It can be stated as follows: (Sharma et al., 2020).


$$\sigma {\left(Z\right)}_{i}=\frac{e^{Z_{i}}}{\sum_{k=1}^{K}e^{Z_{k}}}\;\;\;\;\;\;for\;i=1,2,\ldots ,\mathrm{K.}\;\;$$

In this study, the highest *R* ^2^ and adjusted *R* ^2^ and the lowest RMSE, MAPE, SD ratio, AIC, and corrected Akaike information criterion (AICc) values were achieved using the hyperbolic tangent activation function. The hyperbolic tangent activation function was used because it provided the best prediction.

The output layer will receive the results. Thus, the output layer will obtain the output variable. In the output layer, the linear transfer function (*λ*) is applied as,


$$g=\lambda \left(w_{L}Z+b_{0}\right)$$

where the output layer and the final hidden layer’s connectivity weights are denoted by $w_{L}$ . Furthermore, the output layer bias is *b*_0_ (Rumelhart et al., 1986).

#### 2.2.4 Multivariate adaptive regression spline

Multivariate adaptive regression spline (MARS) is a nonparametric modeling technique that adds nonlinearities and variable interactions to the linear model. This approach is an extension of recursive partitioning regression (RPR), which creates distinct subregions inside the predictor variable space (Friedman, 1991; Montero, 2013). The model is expressed as follows:


$$y_{t}=f\left(x_{t}\right)=\beta_{0}+\sum_{i=1}^{k}\beta_{i}B\left(x_{it}\right)$$

where $\beta_{i}$ , which range from i = 1,…, k, are the model parameters for the corresponding *x_it_* variables and $y_{t}$ is the response variable at instant *t*. The intercept is represented by the value ˇ0, and the basis functions each $B\left(x_{it}\right)$ may be expressed as


$$B\left(x_{it}\right)=\text{max}\left(0,\;x_{it}-c\right)$$

or


$$B\left(x_{it}\right)=\max\left(0,\;c-x_{it}\right),$$

where *c* is a threshold value and *k* is the number of explanatory variables, which includes interactions of the predictor variables (Salford Systems, 2001a). By using only a small number of knots *c*, the MARS algorithm (Friedman, 1991) aims to fit splines of the form $\left(x_{it}-c\right)$ and $\left(c-x_{it}\right),$ to high-dimensional data. Thus, in a forward stepwise fashion, the algorithm looks for the ideal *c* to approximate the relationship between $y_{t}$ and the predictor variables *x_it_*. It begins with an empty model and adds knots to the model recursively for each of the predictor variables in *x_it_*. The variable and knot selected at each phase are chosen to produce the greatest reduction in the final model’s error (Friedman, 1991). For both forms, consider it as a functional value *x_it_*. In the first version, *x_it_* equals $x_{it}-c$ for all values of *x* greater than *c* and is set to 0 for all values of *x_it_* up to a threshold value, *c*. In the second form, *x_it_* equals $c-x_{it}$ for all values of *x* less than *c* and is set to 0 for all values of *x_it_* greater than a threshold value, *c* (Abraham et al., 2001). Every function has a knot at value *c* and is piecewise linear. Linear nonsmooth splines are these transformed functions (Hastie et al., 2009). $B\left(x_{it}\right)$ are functions that rely on the corresponding *x_it_* variables. The data analysis yields the space partition points and model parameters. The complexity of the model is indicated by the number of derived basis functions (Salford Systems, 2001a).

The least squares approach is used to identify the functions with the best estimate performance once the fundamental functions and knots have been identified (Friedman et al., 2001). Generalized crossvalidation (GCV) measurement serves as the basis for model selection (Salford Systems, 2001b).


$$GCV=\sum_{i=1}^{n}\frac{{\left(y_{i}-\hat{y_{i}}\right)}^{2}}{{\left(1-C\left(M\right)/n\right)}^{2}}$$

where *C*(*M*) displays a penalty measure correlated with the number of chosen parameters, and $\hat{y}$ denotes the projected values.

The differences between each method used were displayed in **Table 1**.

**Table 1:** Differences between MARS, CHAID, CHART, and ANN methods.

MARS	A maximum of 35 basis functions was used in the MARS approach. A level − 1 penalty was applied for adding additional basis functions to the model, and interactions between variables were included.
CHAID	The CHAID approach used 10-fold crossvalidation to prevent the model from overlearning. In this method, the input dataset is repeatedly divided into training and test subsets, forming the basis of the model. We can finish the model-building process without overlearning by evaluating their quality at each iteration. When overlearning occurs, the model’s error on the learning set is extremely low.
CART	The CART approach included 10-fold crossvalidation to prevent the model from overlearning. This technique involves repeatedly dividing the input dataset into learning and test subsets, which serve as the foundation for the models. By evaluating their quality at each iteration, we can finish the model-building process without overlearning. When overlearning occurs, the model’s error on the learning set is extremely low.
ANN	The optimal network structure was determined by setting the number of neurons in the hidden layer to 3 in the ANN. A multilayer perceptron model was used in the study.

The following goodness-of-fit criteria were computed in order to compare the prediction performance of the approaches in 10-fold crossvalidation (Willmott and Matsuura, 2005; Takma et al., 2012):

Pearson correlation coefficient (*r*) between the actual and predicted yield (WGY) values,

1. Akaike information criterion (AIC) calculated as:


$$AIC=nln\left[\frac{1}{n}{\left(y_{i}-\hat{y_{i}}\right)}^{2}\right]+2k,\;\;\;if\frac{n}{k}>40$$

or:


$$AIC_{c}=nln\left[\frac{1}{n}{\left(y_{i}-\hat{y}\right)}^{2}\right]+2k+\frac{2k\left(k+1\right)}{n-k-1},\;\;\;\;\;\;\;\;\;\;\;\;\;\;\;\;\;\text{otherwise}$$

2. Root-mean-square error (RMSE) given by the following formula:


$$RMSE=\sqrt{\frac{1}{n}\sum_{i=1}^{n}{\left(y_{i}-\hat{y_{i}}\right)}^{2}}$$

3. Standard deviation ratio (SD_ratio_):


$$SD_{ratio}=\frac{s_{m}}{s_{d}}$$

4. Mean absolute percentage error (MAPE):


$$MAPE=\frac{1}{n}\sum_{i=1}^{n}\left|\frac{y_{i}-\hat{y_{i}}}{y_{i}}\right|.100$$

5. Coefficient of determination


$$R^{2}=1-\frac{\sum_{i=1}^{n}{\left(y_{i}-\hat{y_{i}}\right)}^{2}}{\sum_{i=1}^{n}{\left(y_{i}-{\bar{y}}_{i}\right)}^{2}}$$

6. Adjusted coefficient of determination


$$Adj.R^{2}=1-\frac{\frac{1}{n-k-1}\sum_{i=1}^{n}{\left(y_{i}-\hat{y_{i}}\right)}^{2}}{\frac{1}{n-1}\sum_{i=1}^{n}{\left(y_{i}-{\bar{y}}_{i}\right)}^{2}}$$

where *n* is the number of cases in a set, *k* is the number of model parameters, $y_{i}$ is the output variable’s actual (observed) value, $\hat{y}$ is its predicted value, $s_{m}$ is the standard deviation of model errors, and $s_{d}$ is its output variable’s standard deviation (WGY).

Data mining algorithms are non-parametric statistical techniques that do not require a normality assumption for the dependent variables. These algorithms perform well in cases of missing data for independent variables and can be applied to both large and small datasets (Yordanova et al., 2015).

For algorithms that have varying input numbers, the adjusted coefficient of determination can be utilized as a goodness-of-fit criteria. This adjustment accounts for difference in the number of input variables. The MARS algorithm defines *k* as the number of phrases. IBM SPSS 26 (IBM Corp. Released, 2019) was used for statistical analyses of the CHAID, CART, and ANN algorithms, while the R Studio software (R Core Team, 2022) described the MARS method.

## 3 Results

### 3.1 Descriptive statistics

**Table 2** provides descriptive information about the traits (wet grass yield, crude protein, crude ash, ADF, and NDF) of peas grown in 14 distinct genotypes.

**Table 2:** Descriptive statistics of characteristics of peas.

	Genotype	*N*	Mean	SE	SD	Minimum	Maximum
WGY	88 PO38	3	723.220	77.588	134.387	608.330	871
	SPRING PEA	3	951.560	62.556	108.351	844.330	1061
	P57B	3	723.890	8.957	15.5146	708	739
	P51	3	756.560	108.031	187.114	573.330	947.330
	P101	3	1007	82.924	143.628	860	1147
	P104	3	845.110	71.661	124.121	707	947.330
	ATOS	3	888.560	130.160	225.443	684.670	1,130.670
	ÖZKAYNAK	3	1,003.890	100.561	174.176	851.330	1,193.670
	RETNA	3	1,243.220	121.818	210.995	1,006.670	1,412
	GATEM-101	3	1,178.670	149.953	259.725	879.670	1,348.330
	SPRING	3	735.890	55.690	96.458	659.670	844.330
	BOLERO	3	965.890	10.839	18.774	944.670	980.330
	ÜRÜNLÜ	3	1,273.560	49.998	86.599	1,179.330	1,349.670
	GÖL YAZI	3	1,116.330	85.836	148.673	1,021.330	1,287.670
Crude Protein	88 PO38	3	9.340	0.714	1.237	8.070	10.540
	SPRING PEA	3	9.990	0.242	0.419	9.520	10.330
	P57B	3	9.650	0.649	1.1247	8.620	10.850
	P51	3	11.250	0.762	1.320	10.060	12.670
	P101	3	9.600	0.155	0.269	9.300	9.820
	P104	3	7.640	0.448	0.7766	6.890	8.440
	ATOS	3	11.480	1.142	1.9787	9.360	13.280
	ÖZKAYNAK	3	10.150	0.202	0.350	9.800	10.500
	RETNA	3	10	0.027	0.046	9.950	10.040
	GATEM-101	3	9.630	0.115	0.199	9.410	9.800
	SPRING	3	9.910	0.185	0.320	9.580	10.220
	BOLERO	3	13.810	0.419	0.726	13.100	14.550
	ÜRÜNLÜ	3	11.030	0.598	1.036	9.980	12.050
	GÖL YAZI	3	10.650	0.602	1.042	9.650	11.730
Crude Ash	88 PO38	3	8.310	0.205	0.356	7.970	8.680
	SPRING PEA	3	8.700	0.152	0.263	8.460	8.980
	P57B	3	7.660	0.060	0.104	7.580	7.780
	P51	3	9.410	0.353	0.612	8.880	10.080
	P101	3	7.520	0.022	0.038	7.490	7.560
	P104	3	9.310	0.245	0.424	8.960	9.780
	ATOS	3	8.770	0.095	0.165	8.580	8.870
	ÖZKAYNAK	3	9.660	0.168	0.291	9.380	9.960
	RETNA	3	10.520	0.788	1.365	9.160	11.890
	GATEM-101	3	10.520	0.233	0.403	10.150	10.950
	SPRING	3	12.090	0.032	0.056	12.040	12.150
	BOLERO	3	10.250	0.015	0.025	10.230	10.280
	ÜRÜNLÜ	3	9.810	0.033	0.057	9.760	9.870
	GÖL YAZI	3	8.230	0.257	0.445	7.780	8.670
ADF	88 PO38	3	31.170	0.722	1.250	29.840	32.320
	SPRING PEA	3	30.360	0.866	1.499	28.970	31.950
	P57B	3	34.610	0.789	1.367	33.140	35.840
	P51	3	30.210	0.603	1.045	29.060	31.100
	P101	3	33.650	1.573	2.725	31.020	36.460
	P104	3	35.040	0.489	0.848	34.110	35.770
	ATOS	3	30.510	1.579	2.735	27.800	33.270
	ÖZKAYNAK	3	34.790	0.717	1.242	33.590	36.070
	RETNA	3	28.760	2.020	3.498	26.080	32.720
	GATEM-101	3	33.410	2.116	3.665	29.710	37.040
	SPRING	3	33.400	0.251	0.435	32.960	33.830
	BOLERO	3	27.750	0.026	0.045	27.710	27.800
	ÜRÜNLÜ	3	34.250	1.280	2.217	32.300	36.660
	GÖL YAZI	3	32.560	0.490	0.848	31.760	33.450
NDF	88 PO38	3	41.450	0.556	0.963	40.450	42.370
	SPRING PEA	3	38.040	0.667	1.156	36.820	39.120
	P57B	3	46.030	0.962	1.667	44.460	47.780
	P51	3	41.150	0.420	0.728	40.390	41.840
	P101	3	44.780	1.766	3.059	41.940	48.020
	P104	3	44.260	0.122	0.211	44.020	44.400
	ATOS	3	37.180	2.678	4.638	32.710	41.970
	ÖZKAYNAK	3	43.830	0.285	0.494	43.290	44.260
	RETNA	3	42.080	0.101	0.175	41.910	42.260
	GATEM-101	3	42.870	0.766	1.327	41.660	44.290
	SPRING	3	43.330	0.012	0.020	43.310	43.350
	BOLERO	3	43.970	0.444	0.7696	43.140	44.660
	ÜRÜNLÜ	3	42.090	1.062	1.840	40.180	43.850
	GÖL YAZI	3	40.120	0.240	0.415	39.780	40.580

### 3.2 Results of correlation matrix and principal component analysis

**Figure 1:**
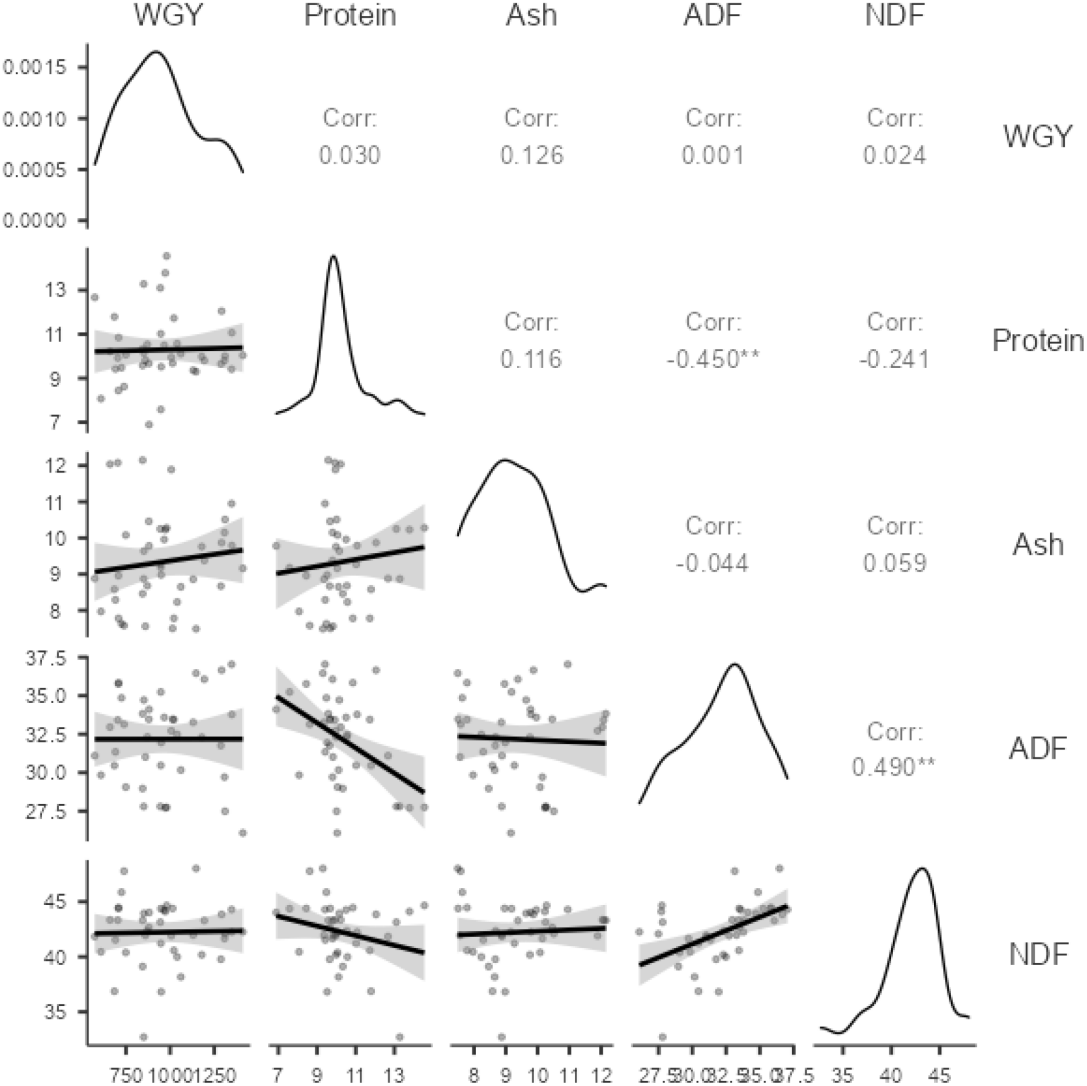
Correlation coefficients between features.

The correlation matrix for the characteristics of peas is presented in **Figure 1**.

**Figure 2:**
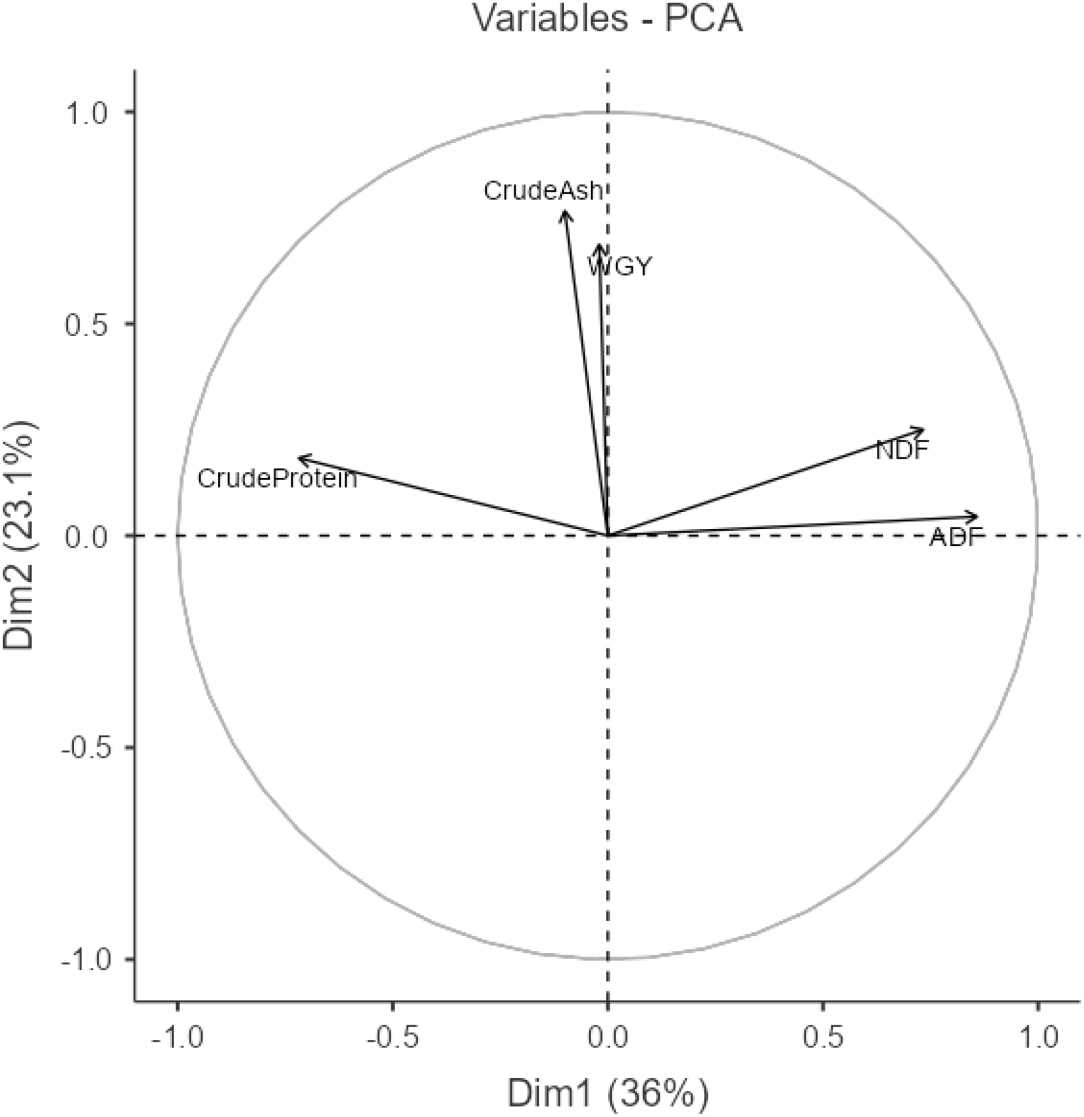
Relationship among traits according to principal component analysis.

When examining the correlation coefficients in **Figure 1**, the highest correlations are observed between the ADF-NDF (0.490) and ADF-crude protein (− 0.450) variables. Correlation coefficients between other traits are low and statistically insignificant. The lowest correlations were between WGY-ADF (0.001), WGY-NDF (0.024), WGY-Protein (0.030), and ADF-Ash (− 0.044), respectively. The representation of the principle component analysis (PCA) graph for the same variables is presented in **Figure 2**.

In PCA analysis, the first principal component (PC1) accounted for 36%, while the second principal component (PC2) accounted for 23.1%. Together, PC1 and PC2 explained a total of 59.1% of the variation. An angle between the slices between 0° and 90° is interpreted as a positive correlation among the traits within those slices, whereas an angle between 90° and 180° is interpreted as a negative association. If the angle is exactly 90°, it indicates no relationship between the traits (Yan and Tinker, 2005: Aktaş, 2017). As the vector moves away from the origin, the variation between variables increases according to the trait examined, whereas the variation decreases as the vector approaches the origin (Abate et al., 2015). Accordingly, the relationship between ADF and NDF is positive. In contrast, the relationships between ADF-WGY and ADF-Crude Ash variables are positive but very weak. Conversely, the relationship between ADF-Crude Protein is negative. While the relationship between NDF and WGY and crude ash variables is positive, the relationship between NDF and crude protein is negative. The relationships between WGY and both crude protein and crude ash are positive. Additionally, the relationship between crude protein and crude ash is also positive.

CHAID, CART, ANN, and MARS techniques were used to investigate the impacts of characteristics on wet grass yield in peas, and the findings are given below.

### 3.3 Result of CHAID algorithm

**Figure 3:**
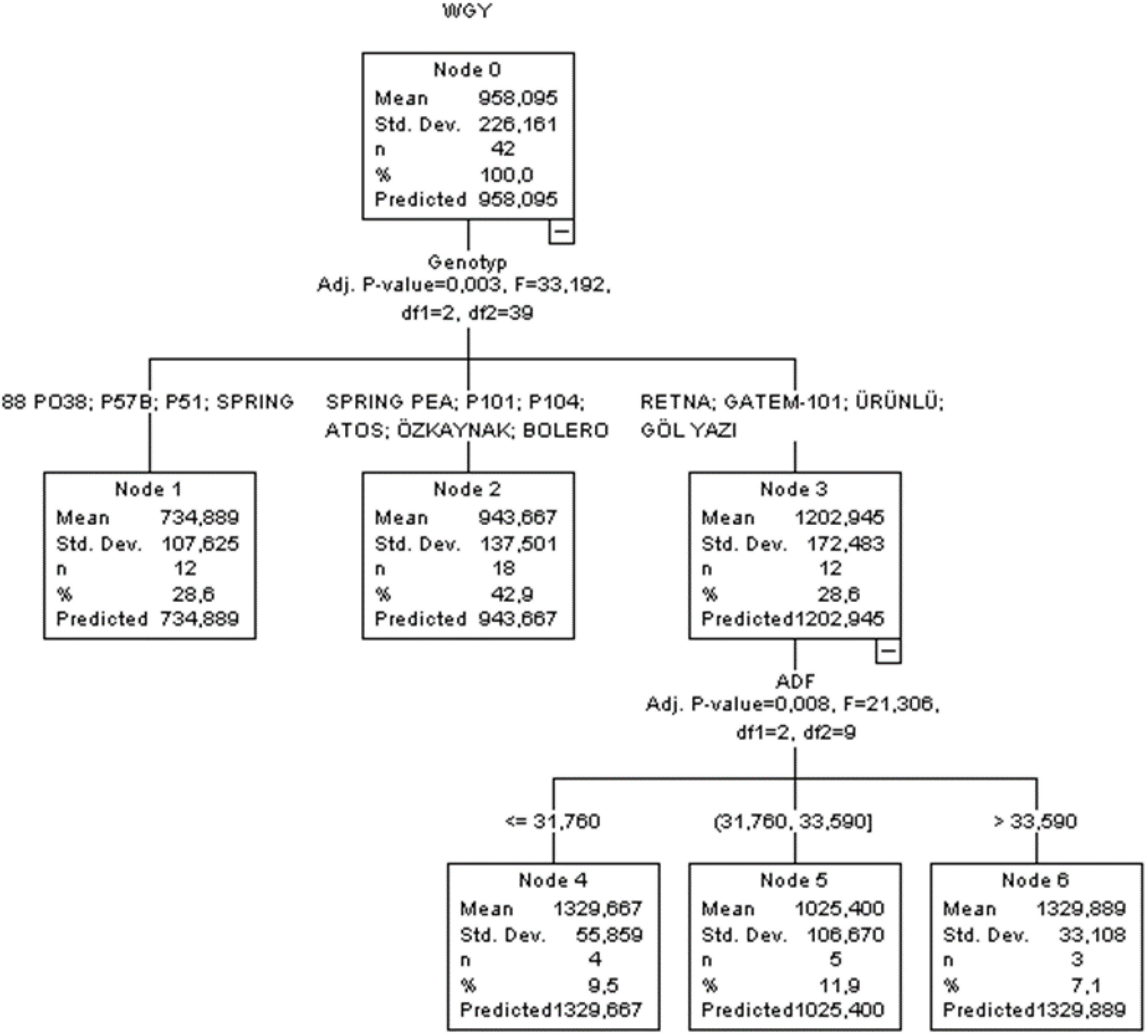
CHAID classification tree diagram of the estimation in wet grass yield.

The CHAID method was used to assess the impacts of various factors on wet grass yield. The parent node to child node ratio was set at 8:4, as this configuration provided better goodness-of-fit criteria within the CHAF algorithm. Crossvalidation was performed with a setting of 10. The regression tree diagram resulting from the CHAID method is shown in **Figure 3**.

An analysis of the CHAID diagram revealed that genotype (Adj. *p*-value = 0.003, *F* = 33.192) was the first-order effective independent variable influencing wet grass yield of peas, followed by ADF (Adj. *p*-value = 0.008, *F* = 21.306) as the second-order variable (**Figure 3**). Throughout the whole tree construction process, the branches produced by independent variables were statistically significant (*p* < 0.05). In terms of *R* ^2^, SD ratio, RMSE, MAPE, AIC, and AICc, the CHAID algorithm’s performance was determined to be 0.759, 0.564, 109.75, 9.198, 443, and 876, respectively. The results of the CHAID algorithm indicated that the highest yield in peas was found to be 1,329.889 kg for the RETNA, GATEM-101, ÜRÜNLÜ, and GÖLYAZI lines, with ADF > 33.59.

### 3.4 Result of CART algorithm

**Figure 4:**
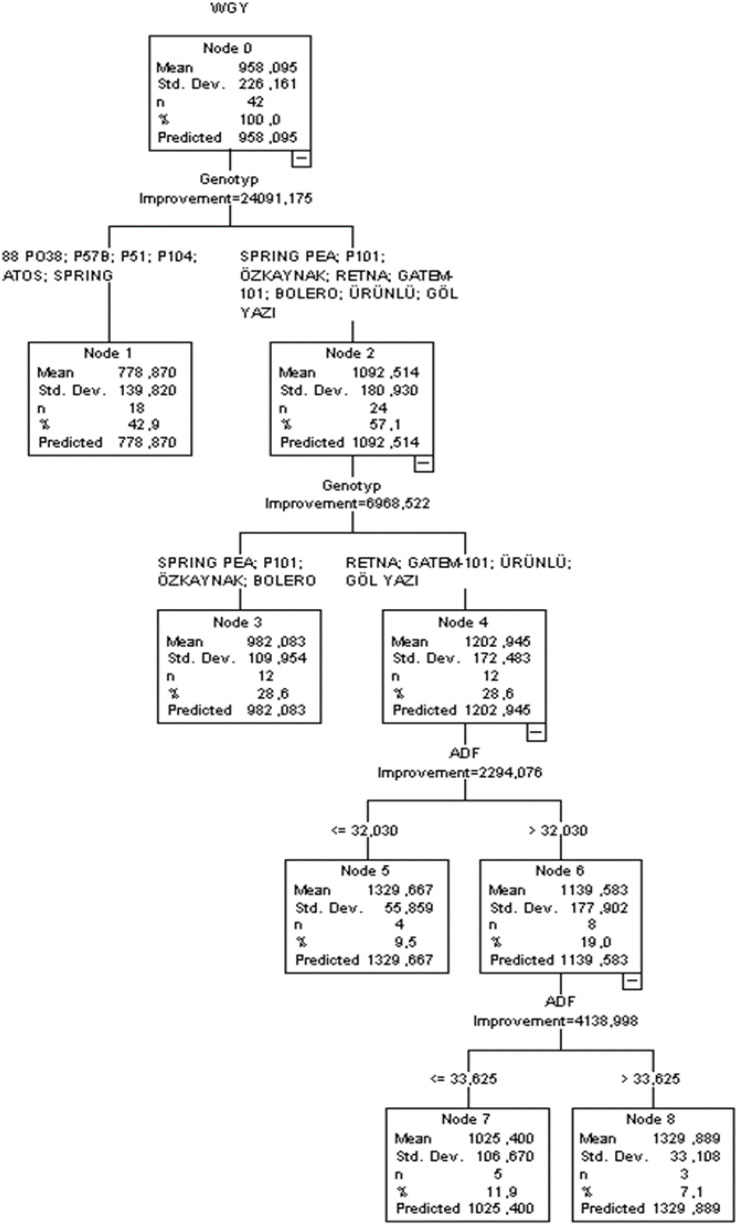
CART classification tree diagram of the estimation in wet grass yield.

The CART technique was used to determine the effects of various variables on wet grass yield. The ratio of parent nodes to child nodes was established at 8 to 4, which resulted in improved goodness-of-fit criteria for the CART algorithm. **Figure 4** illustrates the regression tree diagram generated by the CART algorithm. A crossvalidation approach was set to 10.

The CART diagram revealed that genotype (improvement = 24.091) was the first-order effective independent variable affecting the wet grass yield of peas, closely followed by ADF (improvement = 24.091) (**Figure 4**). Independent factors created significant branches over the tree construction process (*p* < 0.05). The CHAID method performed well in terms of *R* ^2^, SD ratio, RMSE, MAPE, AIC, and AICc, with values of 0.752, 0.576, 111.526, 9.791, 499, and 918, respectively. According to the CART algorithm, the greatest pea yield was recorded at 1,329.889 kg when ADF > 33.625.

### 3.5 Result of artificial neural network

**Figure 5:**
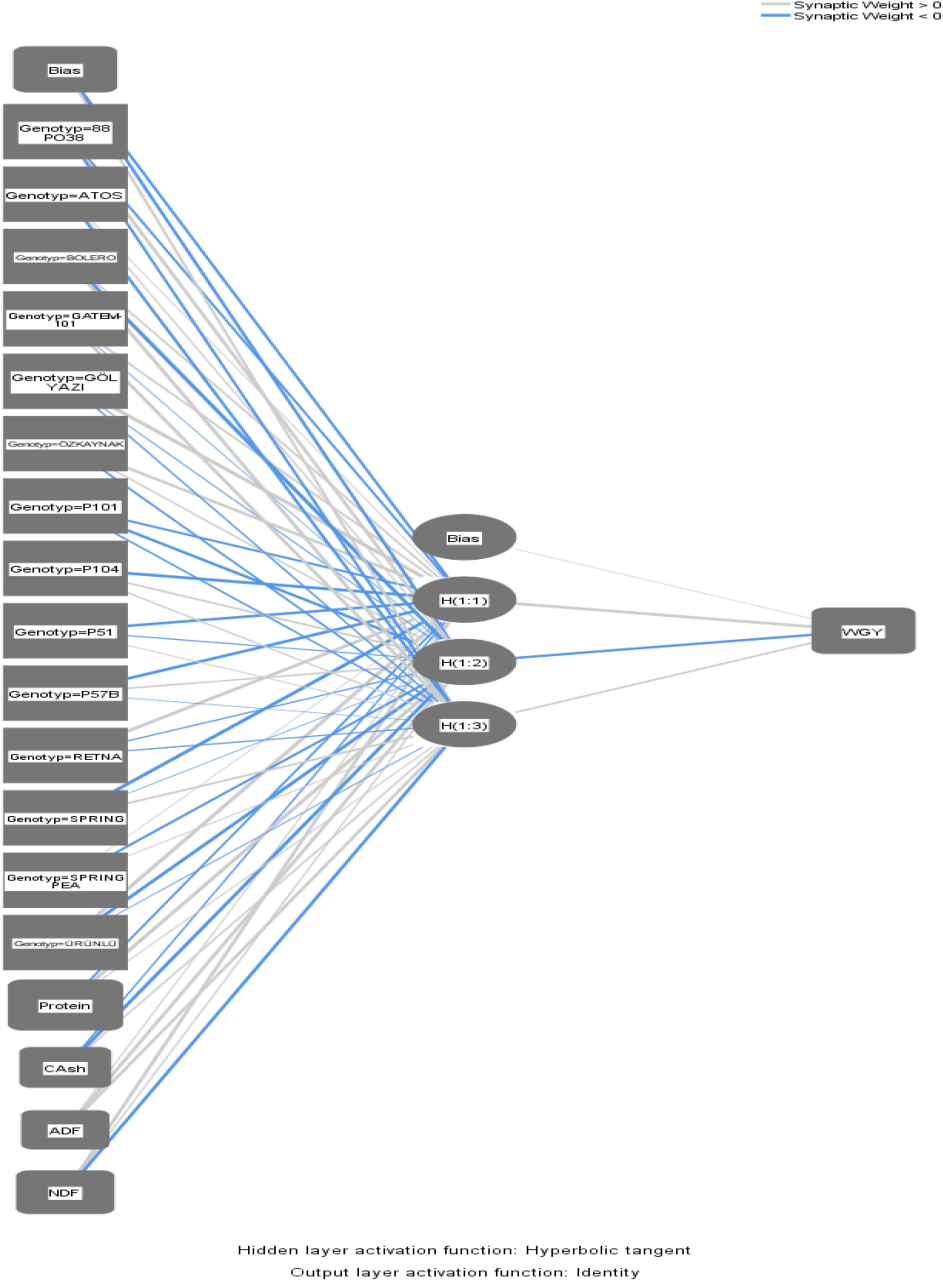
Artificial neural network architecture.

The multilayer perceptron artificial neural network model was chosen for its suitability to the data. A training ratio of 70% and testing ratio of 30% were used, with the scaled conjugate gradient selected as the optimization algorithm. In the present study, an ANN with 10-fold crossvalidation was applied. **Figure 5** shows the connections within the ANN.

In **Figure 5**, the activation function used in the hidden layer of the artificial neural network architecture is the hyperbolic tangent, while the output employs the identity activation function. The parameter estimates of the ANN are presented in **Table 3**.

**Table 3:** Parameter estimates of ANN.

Parameter estimates				
Predictor	Predicted			
	Hidden layer 1		Output layer	
	H(1:1)	H(1:2)	H(1:3)	WGY
Input layer				
(Bias)	− 0.348	− 0.379	0.315	
[Genotype=88 PO38]	− 0.309	0.405	− 0.340	
[Genotype=ATOS]	0.044	0.164	− 0.379	
[Genotype=BOLERO]	0.293	− 0.803	0.472	
[Genotype=GATEM-101]	0.308	− 0.007	0.464	
[Genotype=GÖL YAZI]	1.665	− 0.052	− 0.224	
[Genotype=ÖZKAYNAK]	0.667	0.243	− 0.301	
[Genotype=P101]	− 0.382	− 0.585	− 0.246	
[Genotype=P104]	− 1.514	0.352	0.245	
[Genotype=P51]	− 0.745	− 0.173	0.117	
[Genotype=P57B]	− 1.141	0.315	− 0.003	
[Genotype=RETNA]	1.206	− 0.231	− 0.227	
[Genotype=SPRING]	− 1.068	-0.010	0.355	
[Genotype=SPRING PEA]	0.041	− 0.355	0.081	
[Genotype=ÜRÜNLÜ]	0.827	− 0.685	− 0.045	
Protein	− 0.299	0.699	0.084	
Ash	− 0.193	− 0.577	0.240	
ADF	0.077	0.444	0.389	
NDF	0.427	0.141	− 0.430	
Hidden layer 1				
(Bias)				0.033
H(1:1)				1.264
H(1:2)				− 0.575
H(1:3)				0.320

The connections between each neuron in **Table 3** are as follows:

The connection weight value between protein in the input layer and H(1:1) of the first neuron in the hidden layer is − 0.299. The connection weight value between H(1:2) of the second neuron in the hidden layer is 0.699, while the connection weight value between H(1:3) of the third neuron in the hidden layer is 0.084.

The connection weight value between the ash in the input layer and the H(1:1) of the first neuron in the hidden layer is − 0.193. The connection weight value between the H(1:2) of the second neuron in the hidden layer is − 0.577, and the connection weight value between H(1:3) of the third neuron in the hidden layer is 0.240.

The connection weight value between ADF in the input layer and H(1:1) of the first neuron in the hidden layer is 0.077. For the second neuron in the hidden layer, H(1:2), the connection weight value between is 0.444, while for the third neuron, H(1:3), the connection weight value is 0.389.

The connection weight value between NDF in the input layer and H(1:1) of the first neuron in the hidden layer is 0.427, while H(1:2) of the second neuron has weight of 0.141, and H(1:3) of the third neuron has a weights of − 0.430.

The learning sum of squares error (SSE) in the ANN model was 3.638, with a relative error of 0.269. For the test data, the SSE was 6.577, and the relative error was 0.594.

**Table 4** shows the percentage of importance of independent variables.

**Table 4:** Independent variable importance.

	Importance	Normalized importance
Genotype	0.409	100.00%
Protein	0.317	77.40%
Ash	0.083	20.20%
ADF	0.046	11.30%
NDF	0.145	35.50%

**Figure 6:**
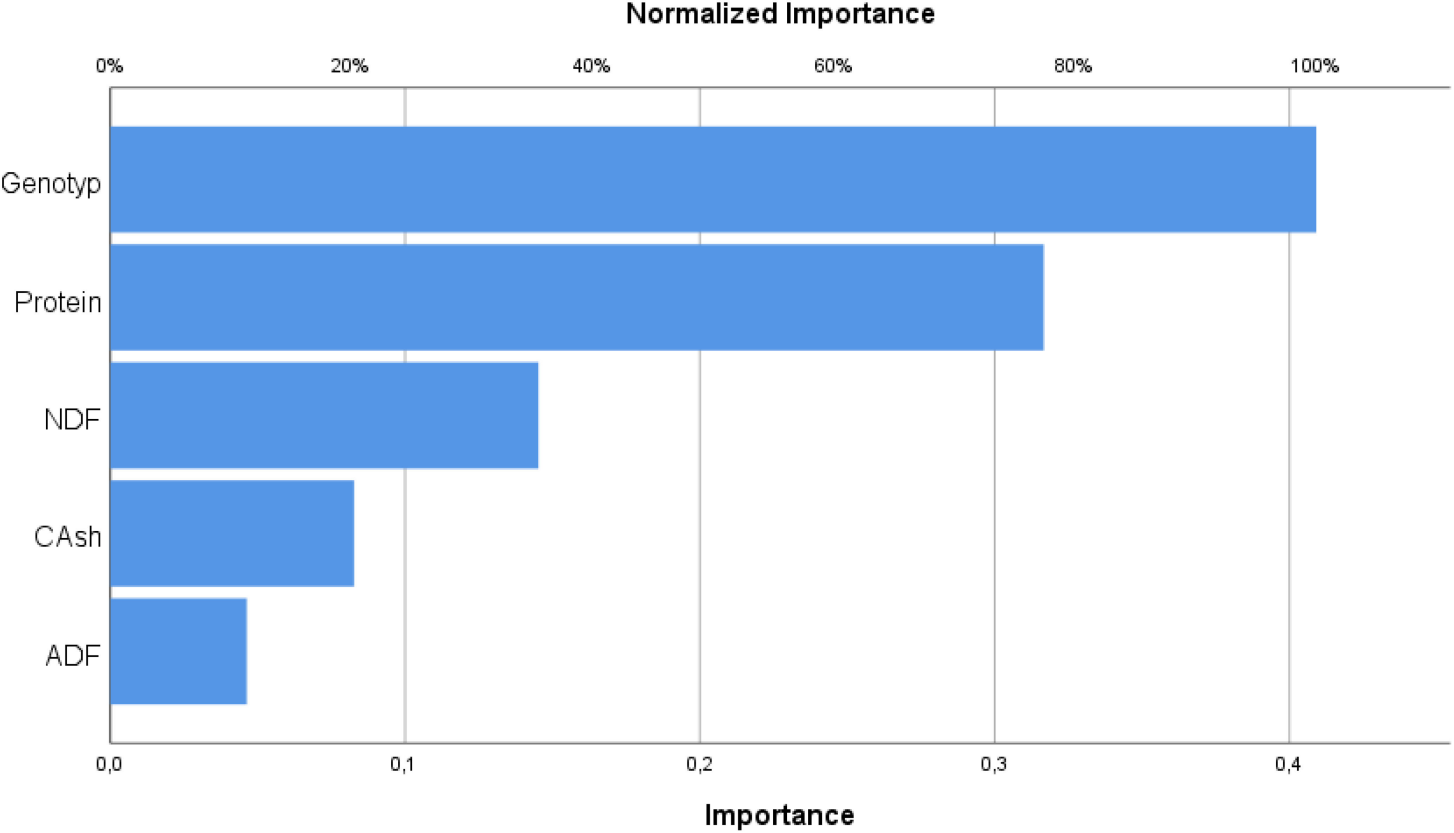
Importance of variables.

As shown in **Table 4**, the independent variables affecting wet grass yield in the output layer include genotype (line) with a coefficient of 0.409, protein at 0.317, ash at 0.083, ADF at 0.046, and NDF at 0.145. **Figure 6** presents a percentage column graph illustrating the influence of these variables on the prediction.

As can be seen in **Table 5**, genotype (line) has the highest influence, accounting for 100% of the effect on the fresh herbage yield of pea plants sold from the terminal on this model. In addition, crude protein is the second most important independent variable with a rate of 77.4%, while NDF accounts for 35.5%. Crude ash has an effect of 20.2%, and ADF has the least impact, with a rate of 11.3% on the fresh herbage yield from the terminals.

**Table 5:** MARS, CHAID, CART, and ANN types’ predictive performance.

	MARS	CHAID	CART	ANN	Decision	The best algorithm
*R* ^2^	0.998	0.759	0.752	0.651	Greater is better	MARS
Adjusted *R* ^2^	0.986	0.747	0.739	0.635	Greater is better	MARS
RMSE	10.499	109.750	111.526	144.009	Smaller is better	MARS
MAPE	0.7365	9.198	9.791	13.589	Smaller is better	MARS
SD ratio	0.047	0.564	0.576	0.922	Smaller is better	MARS
AIC	268	443	499	424	Smaller is better	MARS
AICc	688	876	918	861	Smaller is better	MARS

### 3.6 MARS algorithm results

The model estimation coefficients of the MARS algorithm, which predicts the fresh herbage yield of pea plants, are provided in **Table 6**. A penalty of − 1 penalty and 10-fold crossvalidation were applied in the R studio free software to improve the predictive accuracy of the MARS algorithm.

**Table 6:** Results of MARS algorithm in the prediction of fresh herbage yield of pea plants.

Variables	Coefficients	Std. Error	*t* value	Pr(>|*t*|)
(Intercept)	1,232.876	51.718	23.838	5.81*e*−08^***^
bx[. -1]h(CAsh-9.87)	− 17,220.45	1,717.774	− 10.025	2.10*e*−05^***^
bx[. -1]h(9.87-CAsh)	− 345.434	33.226	− 10.397	1.65*e*−05^***^
bx[. -1]h(Protein-9.67)	− 126.666	11.887	−10.656	1.41*e*−05^***^
bx[. -1]h(9.67-Protein)	− 2,524.694	336.174	−7.51	0.000136^***^
bx[. -1]h(ADF-31.02)	− 118.65	79.948	− 1.484	0.181359
bx[. -1]h(31.02-ADF)	69.189	8.566	8.077	8.57*e*−05^***^
bx[. -1]GenotypGolyazi	−17,608.673	2,884.624	− 6.104	0.000489^***^
bx[. -1]GenotypP51*h(CAsh-9.87)	−1,608.724	218.595	− 7.359	0.000155^***^
bx[. -1]GenotypP101*h(9.87-CAsh)	162.16	12.724	12.745	4.24*e*−06^***^
bx[. -1]GenotypOzkayna	36,677.515	4,457.209	8.229	7.61*e*−05^***^
bx[. -1]GenotypP104	− 29,761.909	6,812.56	− 4.369	0.003279^**^
bx[. -1]GenotypRETNA*h(ADF-31.02)	335.349	69.485	4.826	0.001908^**^
bx[. -1]h(9.67-Protein)*ADF	81.118	10.824	7.494	0.000138^***^
bx[. -1]GenotypUrunlu*h(Protein-9.67)	571.176	104.154	5.484	0.000922^***^
bx[. -1]h(CAsh-9.87)*NDF	384.612	38.595	9.965	2.19e-05^***^
bx[. -1]GenotypOzkayna*NDF	− 1,191.847	151.188	− 7.883	0.000100^***^
bx[. -1]GenotypOzkayna*CAsh	1,575.428	250.733	6.283	0.000411^***^
bx[. -1]GenotypGate101*h(Protein-9.67)	3,143.449	402.036	7.819	0.000105^***^
bx[. -1]GenotypGate101	− 72.392	53.619	− 1.35	0.219005
bx[. -1]GenotypRETNA*h(CAsh-9.87)	668.896	101.938	6.562	0.000315^***^
bx[. -1]GenotypGolyazi*NDF	447.193	71.89	6.221	0.000436^***^
bx[. -1]GenotypUrunlu*h(ADF-31.02)	− 261.754	52.285	− 5.006	0.001554^**^
bx[. -1]GenotypSpring*h(ADF-31.02)	226.808	49.835	4.551	0.002632^**^
bx[. -1]GenotypP104*NDF	651.318	152.853	4.261	0.003742^**^
bx[. -1]GenotypSprinP*h(9.67-Protein)	− 3,131.514	389.113	− 8.048	8.77*e*−05^***^
bx[. -1]h(NDF-41.66)	266.393	77.431	3.44	0.010832^*^
bx[. -1]h(41.66-NDF)	95.905	13.08	7.332	0.000158^***^
bx[. -1]h(NDF-43.14)	− 59.971	39.891	− 1.503	0.176454
bx[. -1]Protein*h(ADF-31.02)	14.653	7.71	1.9	0.099142
bx[. -1]h(NDF-42.08)	− 218.748	95.77	− 2.284	0.056295
bx[. -1]GenotypATOS*h(Protein-9.67)	− 202.012	40.597	− 4.976	0.001608^**^
bx[. -1]GenotypSprinP*h(31.02-ADF)	− 99.857	26.78	− 3.729	0.007370^**^
bx[. -1]GenotypATOS*h(9.87-CAsh)	70.95	29.165	2.433	0.045240^*^
bx[. -1]GenotypGolyazi*h(9.67-Protein)*ADF	142.443	74.291	1.917	0.096704

According to presented results in **Table 4**, all coefficients concerning MARS predictive model were statistically significant (*p* < 0.05, *p* < 0.01, *p* < 0.001). The desirable predictive quality of the MARS equation produced here was obtained with ensuring the smallest GCV (110). The recorded or observed values in fresh herbage yield of pea plants were correlated very strongly with those predicted by the MARS model (*p* < 0.001), indicating effective plant yield modeling. For prediction equation of MARS model with 35 terms, no overfitting problem was recorded, as evidenced by the *R* ^2^ estimate (0.998) being close to the CVR^2^ estimate (0.782). The present SD ratio of 0.047, RMSE of 10.499, MAPE of 0.7365, AIC of 268, and AICc of 688 indicate that the MARS model, which captures influential factors, demonstrates an excellent fit.

According to the MARS method, several terms and coefficients can be read as follows: The equation derived by incorporating the interaction effect of the model’s coefficients is shown in detail below. The effect and corresponding positive coefficient (69.189) on fresh herbage yield were shown to be favorably correlated when ADP ≤ 31.02 in peas; on the other hand, an adverse corresponding negative coefficient (− 126.666) on fresh herbage yield was identified when protein > 9.67. The greatest positive effect on fresh herbage yield in peas was 36,677.515, when the genotype was Özkaynak. The second highest favorable effect, with an increase of 3,143.449, occurred when the genotype was Gate101 and protein > 9.67 have. The third largest positive effect was noted when the genotype was Özkaynak and Cash was present, leading to an increase in fresh herbage yield of 1,575.428.

The greatest negative effect on fresh herbage yield is – 29,761.909 if genotype = P104. The second and third largest negative effects on fresh herbage yield are – 17,608.673 if genotype = Golyazi cm and – 17,220.45 when CAsh > 9.87 cm, respectively.

WGY = 1.23e+03 − 72.4*GenotypGate101 – 17,609*GenotypGolyazi + 36,678*GenotypOzkayna

− 29,762*GenotypP104 – 2,525*max(0, 9.67 − protein) − 127*max(0, protein − 9.67)

− 345*max(0, 9.87 − CAsh) – 17,220*max(0, CAsh − 9.87) + 69.2*max(0, 31 − ADF)

− 119*max(0, ADF − 31) + 95.9*max(0, 41.7 − NDF) + 266*max(0, NDF − 41.7)

− 219*max(0, NDF − 42.1) − 60*max(0, NDF − 43.1) + 447*GenotypGolyazi * NDF

+ 1575*GenotypOzkayna*CAsh – 1,192*GenotypOzkayna*NDF

+ 651*GenotypP104*NDF − 202*GenotypATOS*max(0, protein − 9.67)

+ 71*GenotypATOS*max(0, 9.87 − CAsh) + 3,143*GenotypGate101*max(0, protein − 9.67)

+ 162*GenotypP101*max(0, 9.87 − CAsh) – 1,609*GenotypP51*max(0,CAsh − 9.87)

+ 669*GenotypRETNA*max(0, CAsh − 9.87) + 335*GenotypRETNA*max(0, ADF − 31)

− 3,132*GenotypSprinP*max(0, 9.67− protein) − 99.9*GenotypSprinP*max(0, 31 − ADF)

+ 227*GenotypSpring max(0, ADF − 31) + 571*GenotypUrunlu*max(0, protein − 9.67)

− 262*GenotypUrunlu * max(0, ADF − 31) + 14.7*protein*max(0, ADF − 31)

+ 81.1*max(0, 9.67 − protein)*ADF + 385*max(0, CAsh − 9.87) * NDF

+ 142*GenotypGolyazi*max(0, 9.67 − protein)*ADF

**Figure 7:**
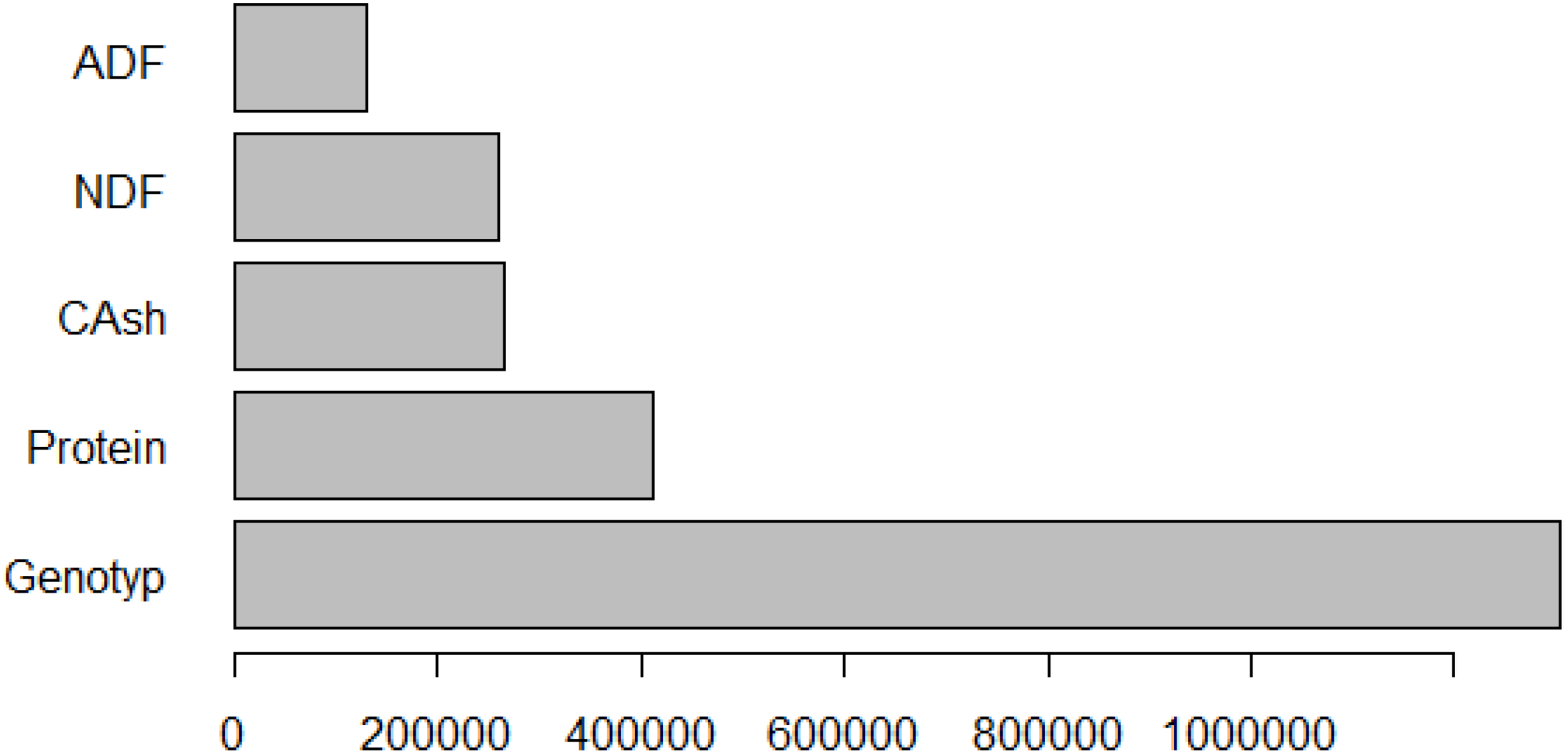
Graph of relative importance for fresh herbage yield.

The relative importance of the variables predicting fresh herbage yield as a result of the MARS algorithm is given in **Figure 7**.

**Figure 8:**
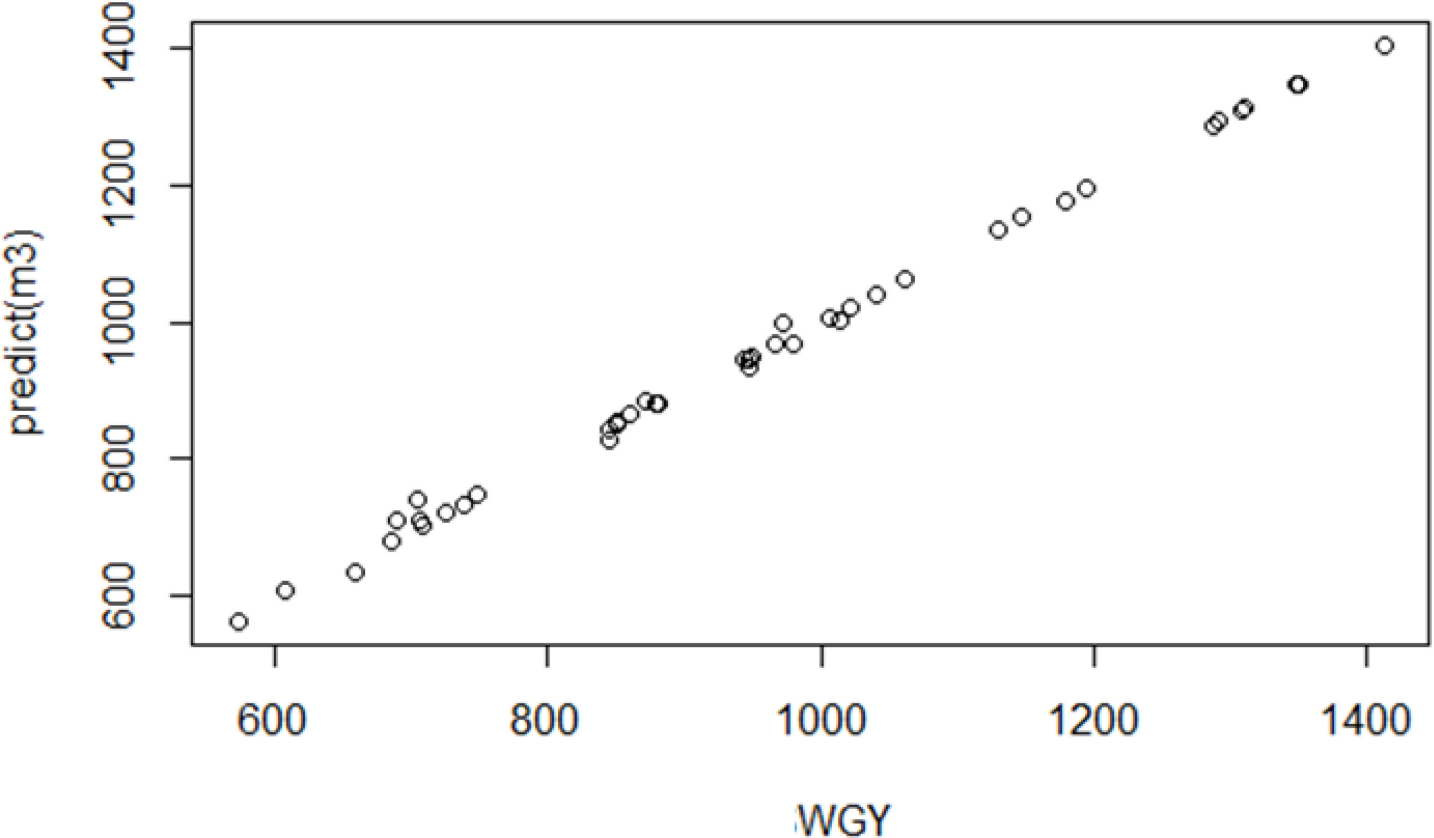
Agreeable expectations and observed fresh herbage yield values.

The graph of the estimated values generated by the MARS algorithm alongside the observed values is shown in **Figure 8**.

Assuming the plant has the following characteristics (genotype = “Özkaynak”, protein = 10.5, Cash = 8, ADF = 32, and NDF = 41.3), the predicted fresh herbage yield of the pea is 992.648 kg.

When predicting fresh herbage production, all algorithms yielded suitable results (**Table 5**). The predicted accuracy of the algorithms was ranked in the following order of superiority: MARS > CHAID > CART > ANN.

*MARS*, multivariate adaptive regression spline; *CHAID*, Chi-square automatic interaction detection; *CART*, classification and regression tree; *ANN*, artificial neural network; *AIC*, Akaike information criterion; *AICc*, corrected Akaike information criterion; *RMSE*, root-mean-square error; *SD*, standard deviation; *MAPE*, mean absolute percentage error; *R^2^*, coefficient of determination.

## 4 Discussion

The average green yield (kg/ha) of pea plants grown in different varieties was found to be between 735.89 and 1,273.56. The crude protein content (%) was 7.64–13.81, crude ash content (%) was 7.52–12.09, NDF content (%) was 37.18–46.03, and ADF content (%) was 27.75–35.04. Notably, the crude protein content observed in this study was lower than the findings of Alatürk et al. (2021) and Uzun et al. (2012), who reported crude protein content percentages of 16.8–20.5 and 9.5–20.4, respectively. The crude ash content in this study was also lower than the findings reported by Alatürk et al. (2021), which ranged from 10.9% to 13.0%. However, NDF and ADF contents of pea forage were in accordance with the study of Alatürk et al. (2021), who reported values of 38.4%–43.2% and 28.6%–34.5%, respectively. In a related study, Çaçan et al. (2018) investigated several forage pea lines and cultivars to determine seed yield, hay yield, and hay quality. They found that seed yield ranged from 33.8 to 180.2 kg ha^−1^, hay yield ranged from 160.3 to 887.0 kg ha^−1^, ash content ranged from 9.42% to 11.19%, crude protein content ranged from 6.54% to 11.91%, crude protein yield ranged from 11.9 to 104.9 kg ha^−1^, ADF ranged from 29.5% to 39.8%, and NDF ranged from 39.1% to 51.2%, respectively. The Gatem, Ürünlü, Gölyazı, and Spring Pea 3-638 genotypes exhibited superior characteristics under Bingöl ecological conditions. In a study by Karadeniz and Bengisu (2022), which investigated the effects of row spacing on yield and quality in green peas (*Pisum sativum* ssp. *arvense*), crude protein was found to range from 20.2% to 22.5%, crude ash from 8.0% to 8.9%, ADF from 32.00% to 33.65%, and NDF from 42.4% to 44.9%. Additionally, a study by Victor (2022) assessed the feed value of green mass of annual legume species and reported the crude protein, ADF, and NDF values of fodder pea as 142 g/kg DM, 392 g/kg DM, and 598 g/kg DM, respectively. Çalık (2020) determined crude protein levels of 10.3%–20.1%, ADF ratios of 21.7%–36.4%, and NDF ratios of 33.2%–43.4% for fodder pea, based on an animal nutrition study assessing various legumes consumed as roughage in Şanlıurfa. In addition, Sarıkaya et al. (2023) conducted a study to determine the effects of different sowing times and plant densities on dry grass yield and quality in some forage pea varieties, finding average values of 14.16% for crude protein, 27.98% for ADF, and 37.64% for NDF. Kara and Sürmen (2023) examined eight different forage pea cultivars (Kirazlı, Ulubatlı, Ürünlü, Gölyazı, Özkaynak, Töre, Taşkent, GAP Pembesi) and subjected them to mowing treatments in three different phenological periods (10%, 50%, and 100% flowering) under Aydın ecological conditions, finding crude protein levels of 19.88%, ADF ratios of 32.57%, and NDF ratios of 45.25%. In a separate study, Kır (2022) investigated the appropriate mixing ratios of fodder pea (*Pisum sativum* ssp. *arvense* L.) and rye (*Secale cereale* L.) under rainy conditions in Kırşehir province during the 2018–2019 vegetation period, reporting crude protein levels of 15.6%, ADF ratios of 31.1%, and NDF ratios of 39.3%.

In the study of Çelik et al. (2024), the factors influencing fresh herbage yield in sorghum–sudangrass hybrid plants were analyzed using CHAID, CART, MARS, and Bagging MARS algorithms. MARS, Bagging MARS, CART, and CHAID were found to be the most appropriate algorithms for predicting the dependent variable. In this study, the MARS algorithm emerged as the best predictor of crop yield, followed by CHAID, CART and ANN methods, respectively. As in the authors’ study, the CART algorithm ranked as the third best method in this study. The *R* ^2^, adjusted *R* ^2^, and SD ratio statistics were closely aligned in both this study and the authors’ research.

The findings obtained of this study show some similarities and some differences with those of previously mentioned researchers. These differences are likely attributed to a combination of factors, with one of the most significant being the variations in climate and soil structures among the study areas. Different climatic and soil conditions can directly affect the development of vegetation and the application of agricultural practices. This can lead to differences in the findings. Another factor is the different practices used by different researchers (such as different fertilization methods, irrigation techniques, weed control methods, and harvest times).

## 5 Conclusion

In this study, the performances of CHAID, CART, ANN, and MARS methods were analyzed to predict wet grass yield in pea plants. The input variables included genotype (line), crude protein (%), crude ash (%), ADF (%), and NDF (%). The results were compared using different goodness-of-fit tests, including the coefficient of determination (*R* ^2^), adjusted *R* ^2^, RMSE, MAPE, SD ratio, AIC, and AICc. The results of this study are presented below.

According to the results of the MARS algorithm, the variables that contributed the most to wet herbage yield in pea plants were genotype, crude protein, crude ash, NDF and ADF. As a result of the application of artificial neural network method, the order of importance of the variables affecting wet grass yield in pea was identified as genotype, crude protein, NDF, crude ash, and ADF. The CHAID algorithm estimated the highest fresh herbage yield of pea at 1,329.889 kg in RETNA, GATEM-101, ÜRÜNLÜ, and GÖLYAZI lines, with ADF > 33.59. When the CART algorithm was applied, the highest herbage yield was reached when ADF > 33.625, resulting in an estimated yield of 1,329.889 kg. In this case, the results from the CHAID and CART algorithms were very close to each other. The performance findings are as follows: MARS > CHAID > CART > ANN (best to worst).

It was determined that mining approaches are quite effective in field agricultural data for identifying factors influencing plant production and predicting any variables.

## Data Availability

Publicly available datasets were analyzed in this study. For more information on the original contributions presented in this study, please contact the corresponding authors.
